# Supplementary material for: Emergence of syntax and word prediction in an artificial neural circuit of the cerebellum
Source: Nat Commun. 2024 Jan 31;15:927. doi: 10.1038/s41467-024-44801-6 (PMC10831061; doi:10.1038/s41467-024-44801-6)
Supplement: Supplementary file 2 — Reporting Summary [file 41467_2024_44801_MOESM2_ESM.pdf]

nature portfolio

Corresponding author(s): Shogo Ohmae

Last updated by author(s): Dec 5, 2023

Reporting Summary

Nature Portfolio wishes to improve the reproducibility of the work that we publish. This form provides structure for consistency and transparency in reporting. For further information on Nature Portfolio policies, see our Editorial Policies and the Editorial Policy Checklist.

Statistics

For all statistical analyses, confirm that the following items are present in the figure legend, table legend, main text, or Methods section.

n/a	Confirmed	
☐	☒	The exact sample size (*n*) for each experimental group/condition, given as a discrete number and unit of measurement
☒	☐	A statement on whether measurements were taken from distinct samples or whether the same sample was measured repeatedly
☐	☒	The statistical test(s) used AND whether they are one- or two-sided<br>*Only common tests should be described solely by name; describe more complex techniques in the Methods section.*
☒	☐	A description of all covariates tested
☒	☐	A description of any assumptions or corrections, such as tests of normality and adjustment for multiple comparisons
☐	☒	A full description of the statistical parameters including central tendency (e.g. means) or other basic estimates (e.g. regression coefficient) AND variation (e.g. standard deviation) or associated estimates of uncertainty (e.g. confidence intervals)
☐	☒	For null hypothesis testing, the test statistic (e.g. *F*, *t*, *r*) with confidence intervals, effect sizes, degrees of freedom and *P* value noted<br>*Give P values as exact values whenever suitable.*
☒	☐	For Bayesian analysis, information on the choice of priors and Markov chain Monte Carlo settings
☒	☐	For hierarchical and complex designs, identification of the appropriate level for tests and full reporting of outcomes
☒	☐	Estimates of effect sizes (e.g. Cohen's *d*, Pearson's *r*), indicating how they were calculated

Our web collection on statistics for biologists contains articles on many of the points above.

Software and code

Policy information about availability of computer code

Data collection	To develop the cerebellar ANN model, we created custom Python code which uses Google's TensorFlow and Keras framework (version 2.9 and 2.11). The code is accessible at: https://github.com/cANN-NLP/NLP_codes (DOI 10.5281/zenodo.10257296).
Data analysis	For visualizing and plotting data, we primarily employed standard Python functions (version 3.10). Additionally, for analysis using machine learning, we utilized the Python library scikit-learn (ver 1.2). To assess the performance of the cANNs in next-word prediction and syntactic processing after training, we used relatively simple sentences from online resources of SentenceDict, Lengusa, and Rong-Chang (https://sentencedict.com/; https://lengusa.com/; https://www.rong-chang.com/nse/).

For manuscripts utilizing custom algorithms or software that are central to the research but not yet described in published literature, software must be made available to editors and reviewers. We strongly encourage code deposition in a community repository (e.g. GitHub). See the Nature Portfolio guidelines for submitting code & software for further information.

Data

Policy information about availability of data

All manuscripts must include a data availability statement. This statement should provide the following information, where applicable:

- Accession codes, unique identifiers, or web links for publicly available datasets
- A description of any restrictions on data availability
- For clinical datasets or third party data, please ensure that the statement adheres to our policy

The simulation data generated in this study are available on Open Science Framework repository (https://osf.io/bwpnk/?view_only=7a9ae0981c2e4f8a921161dda9d079bd).
To train the cANNs, we used 88711 sentences sourced from the Gutenberg Corpus (novels such as Moby-Dick, Alice in Wonderland, etc.) and the Brown Corpus of the Natural Language Toolkit (NLTK, version 3.8). To evaluate the performance of the cANNs at next-word prediction and syntactic processing after training, we used relatively simple sentences from the online resources of SentenceDict, Lengusa, and Rong-Chang (https://sentencedict.com/; https://lengusa.com/; https://www.rong-chang.com/nse/). The source data of the figures are available as a source data file in the Supplementary Material.

Research involving human participants, their data, or biological material

Policy information about studies with human participants or human data. See also policy information about sex, gender (identity/presentation), and sexual orientation and race, ethnicity and racism.

Reporting on sex and gender	Not applicable
Reporting on race, ethnicity, or other socially relevant groupings	Not applicable
Population characteristics	Not applicable
Recruitment	Not applicable
Ethics oversight	Not applicable

Note that full information on the approval of the study protocol must also be provided in the manuscript.

Field-specific reporting

Please select the one below that is the best fit for your research. If you are not sure, read the appropriate sections before making your selection.

☒ Life sciences ☐ Behavioural & social sciences ☐ Ecological, evolutionary & environmental sciences

For a reference copy of the document with all sections, see nature.com/documents/nr-reporting-summary-flat.pdf

Life sciences study design

All studies must disclose on these points even when the disclosure is negative.

Sample size	For data analysis, we had each cANN to make predictions for 1328 words in the evaluation sentences. The ANNs were independently created (initialized) and trained. The number of ANNs ('n' number) was 20 for the original cANN, 10 for the convergent cANN, and 4 to 8 for the other cANN variants. The size of 'n' number was determined according to prior papers (1 to 30, with the majority having only one; Yamazaki and Tanaka 2007; Egger, Le, and Jazayeri 2020; Gilmer et al. 2023; Litwin-Kumar et al. 2017). Additionally, the observed variability in the simulations was consistently minimal, indicating that these sample sizes are adequate for reproducing results.
Data exclusions	No simulated data was excluded.
Replication	The simulation code was thoroughly tested for bugs. Consequently, there were no results that could not be reproduced.
Randomization	In each simulation, synaptic connectivity matrices were randomly initialized by standard initializers. No random allocation of samples between groups was performed as we ensured that exactly even samples were allocated.
Blinding	No blinding was performed because the Python analysis code treated simulation results equally across all groups, ensuring unbiased treatment.

Reporting for specific materials, systems and methods

We require information from authors about some types of materials, experimental systems and methods used in many studies. Here, indicate whether each material, system or method listed is relevant to your study. If you are not sure if a list item applies to your research, read the appropriate section before selecting a response.

Materials & experimental systems

n/a	Involved in the study
☒	☐ Antibodies
☒	☐ Eukaryotic cell lines
☒	☐ Palaeontology and archaeology
☒	☐ Animals and other organisms
☒	☐ Clinical data
☒	☐ Dual use research of concern
☒	☐ Plants

Methods

n/a	Involved in the study
☒	☐ ChIP-seq
☒	☐ Flow cytometry
☒	☐ MRI-based neuroimaging
